# Supplementary material for: Transcriptome Analysis Reveals Vimentin-Induced Disruption of Cell–Cell Associations Augments Breast Cancer Cell Migration
Source: Cells. 2022 Dec 13;11(24):4035. doi: 10.3390/cells11244035 (PMC9776984; doi:10.3390/cells11244035)
Supplement: Supplementary file 1 [file cells-11-04035-s001.zip › DEGs and enrichment analysis.zip!/DEGs and enrichment analysis/WT_VIMvsVector KEGG/ALL/WT_VIMvsVector_ALL.pathway_map.zip!/WT_VIMvsVector_ALL.pathway_map/WT_VIMvsVector_all_KEGGenrich.xls_rendered_html_detail.html]

Pathway Enrichment


  

# The most enriched pathway terms

Statistic method: hypergeometric test

FDR correction method: Benjamini and Hochberg

| Term | Description | Sample number | Background number | P-value | Corrected P-value | Gene\_id | Gene\_name | KEGG\_ID |
| --- | --- | --- | --- | --- | --- | --- | --- | --- |
| hsa03030 | DNA replication | 9\|253 | 41\|9228 | 1.23477977244039e-06 | 0.000321042740834501 | ENSG00000073111 ENSG00000100297 ENSG00000166508 ENSG00000168496 ENSG00000049541 ENSG00000062822 ENSG00000106628 ENSG00000014138 ENSG00000198056 | MCM2 MCM5 MCM7 FEN1 RFC2 POLD1 POLD2 POLA2 PRIM1 | hsa:4171 hsa:4174 hsa:4176 hsa:2237 hsa:5982 hsa:5424 hsa:5425 hsa:23649 hsa:5557 |
| hsa04110 | Cell cycle | 16\|253 | 163\|9228 | 9.60174040167418e-06 | 0.00124822625221764 | novel.507 ENSG00000093009 ENSG00000127564 ENSG00000166851 ENSG00000117399 ENSG00000073111 ENSG00000092969 ENSG00000085840 ENSG00000135476 ENSG00000100297 ENSG00000166508 ENSG00000101412 ENSG00000145386 ENSG00000164045 ENSG00000157456 ENSG00000116670 | - CDC45 PKMYT1 PLK1 CDC20 MCM2 TGFB2 ORC1 ESPL1 MCM5 MCM7 E2F1 CCNA2 CDC25A CCNB2 MAD2L2 | hsa:1870 hsa:8318 hsa:9088 hsa:5347 hsa:991 hsa:4171 hsa:7042 hsa:4998 hsa:9700 hsa:4174 hsa:4176 hsa:1869 hsa:890 hsa:993 hsa:9133 hsa:10459 |
| hsa05146 | Amoebiasis | 11\|253 | 94\|9228 | 4.8893483224674e-05 | 0.00423743521280508 | ENSG00000169429 ENSG00000058085 ENSG00000092969 ENSG00000188153 ENSG00000169031 ENSG00000164742 ENSG00000081041 ENSG00000135862 ENSG00000053747 ENSG00000137462 ENSG00000168542 | CXCL8 LAMC2 TGFB2 COL4A5 COL4A3 ADCY1 CXCL2 LAMC1 LAMA3 TLR2 COL3A1 | hsa:3576 hsa:3918 hsa:7042 hsa:1287 hsa:1285 hsa:107 hsa:2920 hsa:3915 hsa:3909 hsa:7097 hsa:1281 |
| hsa05222 | Small cell lung cancer | 11\|253 | 121\|9228 | 0.000471573482868652 | 0.0306522763864624 | novel.507 ENSG00000005961 ENSG00000058085 ENSG00000188153 ENSG00000169031 ENSG00000101412 ENSG00000171791 ENSG00000173207 ENSG00000135862 ENSG00000053747 ENSG00000023445 | - ITGA2B LAMC2 COL4A5 COL4A3 E2F1 BCL2 CKS1B LAMC1 LAMA3 BIRC3 | hsa:1870 hsa:3674 hsa:3918 hsa:1287 hsa:1285 hsa:1869 hsa:596 hsa:1163 hsa:3915 hsa:3909 hsa:330 |
| hsa04080 | Neuroactive ligand-receptor interaction | 14\|253 | 219\|9228 | 0.00279012617513493 | 0.145086561107017 | ENSG00000078401 ENSG00000164128 ENSG00000135346 ENSG00000136928 ENSG00000187730 ENSG00000109255 ENSG00000168539 ENSG00000069482 ENSG00000148926 ENSG00000168398 ENSG00000171631 ENSG00000065325 ENSG00000180739 ENSG00000164418 | EDN1 NPY1R CGA GABBR2 GABRD NMU CHRM1 GAL ADM BDKRB2 P2RY6 GLP2R S1PR5 GRIK2 | hsa:1906 hsa:4886 hsa:1081 hsa:9568 hsa:2563 hsa:10874 hsa:1128 hsa:51083 hsa:133 hsa:624 hsa:5031 hsa:9340 hsa:53637 hsa:2898 |
| hsa05161 | Hepatitis B | 12\|253 | 179\|9228 | 0.00375161775971133 | 0.162570102920824 | novel.507 ENSG00000157613 ENSG00000179388 ENSG00000169429 ENSG00000092969 ENSG00000138378 ENSG00000089685 ENSG00000101412 ENSG00000171791 ENSG00000145386 novel.600 ENSG00000137462 | - CREB3L1 EGR3 CXCL8 TGFB2 STAT4 BIRC5 E2F1 BCL2 CCNA2 - TLR2 | hsa:1870 hsa:90993 hsa:1960 hsa:3576 hsa:7042 hsa:6775 hsa:332 hsa:1869 hsa:596 hsa:890 hsa:1388 hsa:7097 |
| hsa04218 | Cellular senescence | 12\|253 | 204\|9228 | 0.0103704478240336 | 0.303714213416405 | novel.507 ENSG00000169429 ENSG00000146674 ENSG00000111206 ENSG00000101057 ENSG00000162909 ENSG00000092969 ENSG00000101412 ENSG00000145386 ENSG00000164045 ENSG00000185650 ENSG00000157456 | - CXCL8 IGFBP3 FOXM1 MYBL2 CAPN2 TGFB2 E2F1 CCNA2 CDC25A ZFP36L1 CCNB2 | hsa:1870 hsa:3576 hsa:3486 hsa:2305 hsa:4605 hsa:824 hsa:7042 hsa:1869 hsa:890 hsa:993 hsa:677 hsa:9133 |
| hsa04926 | Relaxin signaling pathway | 9\|253 | 133\|9228 | 0.010802934442159 | 0.303714213416405 | ENSG00000078401 ENSG00000157613 ENSG00000127588 ENSG00000188153 ENSG00000169031 ENSG00000137745 ENSG00000164742 novel.600 ENSG00000168542 | EDN1 CREB3L1 GNG13 COL4A5 COL4A3 MMP13 ADCY1 - COL3A1 | hsa:1906 hsa:90993 hsa:51764 hsa:1287 hsa:1285 hsa:4322 hsa:107 hsa:1388 hsa:1281 |
| hsa04115 | p53 signaling pathway | 7\|253 | 89\|9228 | 0.010947047211653 | 0.303714213416405 | ENSG00000146674 ENSG00000177519 ENSG00000171791 ENSG00000075218 ENSG00000206075 ENSG00000184990 ENSG00000157456 | IGFBP3 RPRM BCL2 GTSE1 SERPINB5 SIVA1 CCNB2 | hsa:3486 hsa:56475 hsa:596 hsa:51512 hsa:5268 hsa:10572 hsa:9133 |
| hsa04512 | ECM-receptor interaction | 7\|253 | 91\|9228 | 0.0122837005437132 | 0.303714213416405 | ENSG00000005961 ENSG00000058085 ENSG00000188153 ENSG00000169031 ENSG00000135862 ENSG00000053747 ENSG00000115221 | ITGA2B LAMC2 COL4A5 COL4A3 LAMC1 LAMA3 ITGB6 | hsa:3674 hsa:3918 hsa:1287 hsa:1285 hsa:3915 hsa:3909 hsa:3694 |
| hsa05206 | MicroRNAs in cancer | 11\|253 | 185\|9228 | 0.0128494474906941 | 0.303714213416405 | ENSG00000026025 novel.507 ENSG00000092969 ENSG00000101412 ENSG00000171791 ENSG00000146670 ENSG00000164045 ENSG00000206075 ENSG00000277443 ENSG00000156103 ENSG00000088305 | VIM - TGFB2 E2F1 BCL2 CDCA5 CDC25A SERPINB5 MARCKS MMP16 DNMT3B | hsa:7431 hsa:1870 hsa:7042 hsa:1869 hsa:596 hsa:113130 hsa:993 hsa:5268 hsa:4082 hsa:4325 hsa:1789 |
| hsa04151 | PI3K-Akt signaling pathway | 18\|253 | 376\|9228 | 0.0152242659779366 | 0.329859096188627 | ENSG00000005961 ENSG00000157613 ENSG00000160867 ENSG00000058085 ENSG00000128052 ENSG00000127588 ENSG00000109321 ENSG00000188153 ENSG00000168539 ENSG00000169031 ENSG00000171791 novel.600 ENSG00000135862 ENSG00000053747 ENSG00000115221 ENSG00000037280 ENSG00000137462 ENSG00000118513 | ITGA2B CREB3L1 FGFR4 LAMC2 KDR GNG13 AREG COL4A5 CHRM1 COL4A3 BCL2 - LAMC1 LAMA3 ITGB6 FLT4 TLR2 MYB | hsa:3674 hsa:90993 hsa:2264 hsa:3918 hsa:3791 hsa:51764 hsa:374 hsa:1287 hsa:1128 hsa:1285 hsa:596 hsa:1388 hsa:3915 hsa:3909 hsa:3694 hsa:2324 hsa:7097 hsa:4602 |
| hsa04914 | Progesterone-mediated oocyte maturation | 8\|253 | 120\|9228 | 0.0172005525250261 | 0.344011050500521 | ENSG00000082175 ENSG00000127564 ENSG00000166851 ENSG00000145386 ENSG00000164045 ENSG00000164742 ENSG00000157456 ENSG00000116670 | PGR PKMYT1 PLK1 CCNA2 CDC25A ADCY1 CCNB2 MAD2L2 | hsa:5241 hsa:9088 hsa:5347 hsa:890 hsa:993 hsa:107 hsa:9133 hsa:10459 |
| hsa00830 | Retinol metabolism | 5\|253 | 57\|9228 | 0.0195688740429394 | 0.363421946511733 | ENSG00000162496 ENSG00000128918 ENSG00000003137 ENSG00000256612 ENSG00000095596 | DHRS3 ALDH1A2 CYP26B1 CYP2B7P CYP26A1 | hsa:9249 hsa:8854 hsa:56603 hsa:1555 hsa:1592 |
| hsa04933 | AGE-RAGE signaling pathway in diabetic complications | 7\|253 | 102\|9228 | 0.0218015565281237 | 0.377893646487478 | ENSG00000078401 ENSG00000169429 ENSG00000092969 ENSG00000188153 ENSG00000169031 ENSG00000171791 ENSG00000168542 | EDN1 CXCL8 TGFB2 COL4A5 COL4A3 BCL2 COL3A1 | hsa:1906 hsa:3576 hsa:7042 hsa:1287 hsa:1285 hsa:596 hsa:1281 |
| hsa04974 | Protein digestion and absorption | 6\|253 | 85\|9228 | 0.0289283819323285 | 0.441762786559584 | ENSG00000092068 ENSG00000188153 ENSG00000164626 ENSG00000169031 ENSG00000104783 ENSG00000168542 | SLC7A8 COL4A5 KCNK5 COL4A3 KCNN4 COL3A1 | hsa:23428 hsa:1287 hsa:8645 hsa:1285 hsa:3783 hsa:1281 |
| hsa00340 | Histidine metabolism | 3\|253 | 25\|9228 | 0.0300150087443358 | 0.441762786559584 | ENSG00000259583 ENSG00000184254 ENSG00000137124 | AC015712.2 ALDH1A3 ALDH1B1 | hsa:220 hsa:220 hsa:219 |
| hsa00982 | Drug metabolism - cytochrome P450 | 5\|253 | 64\|9228 | 0.0305835775310482 | 0.441762786559584 | ENSG00000213366 ENSG00000259583 ENSG00000184254 ENSG00000256612 ENSG00000241720 | GSTM2 AC015712.2 ALDH1A3 CYP2B7P AL158847.1 | hsa:2946 hsa:220 hsa:220 hsa:1555 hsa:2947 |
| hsa04928 | Parathyroid hormone synthesis, secretion and action | 7\|253 | 113\|9228 | 0.0355174470909223 | 0.475350161991246 | ENSG00000157613 ENSG00000137745 ENSG00000171791 ENSG00000164742 novel.600 ENSG00000109062 ENSG00000156103 | CREB3L1 MMP13 BCL2 ADCY1 - SLC9A3R1 MMP16 | hsa:90993 hsa:4322 hsa:596 hsa:107 hsa:1388 hsa:9368 hsa:4325 |
| hsa00240 | Pyrimidine metabolism | 5\|253 | 68\|9228 | 0.0383314959020783 | 0.475350161991246 | ENSG00000168032 ENSG00000167900 ENSG00000128951 ENSG00000176890 ENSG00000168393 | ENTPD3 TK1 DUT TYMS DTYMK | hsa:956 hsa:7083 hsa:1854 hsa:7298 hsa:1841 |
| hsa00980 | Metabolism of xenobiotics by cytochrome P450 | 5\|253 | 69\|9228 | 0.0404393725351685 | 0.475350161991246 | ENSG00000213366 ENSG00000259583 ENSG00000184254 ENSG00000256612 ENSG00000241720 | GSTM2 AC015712.2 ALDH1A3 CYP2B7P AL158847.1 | hsa:2946 hsa:220 hsa:220 hsa:1555 hsa:2947 |
| hsa04510 | Focal adhesion | 12\|253 | 249\|9228 | 0.0413073449200678 | 0.475350161991246 | ENSG00000005961 ENSG00000058085 ENSG00000128052 ENSG00000162909 ENSG00000188153 ENSG00000169031 ENSG00000171791 ENSG00000135862 ENSG00000053747 ENSG00000023445 ENSG00000115221 ENSG00000037280 | ITGA2B LAMC2 KDR CAPN2 COL4A5 COL4A3 BCL2 LAMC1 LAMA3 BIRC3 ITGB6 FLT4 | hsa:3674 hsa:3918 hsa:3791 hsa:824 hsa:1287 hsa:1285 hsa:596 hsa:3915 hsa:3909 hsa:330 hsa:3694 hsa:2324 |
| hsa04621 | NOD-like receptor signaling pathway | 10\|253 | 195\|9228 | 0.0420502066376872 | 0.475350161991246 | ENSG00000169429 ENSG00000117226 ENSG00000162645 ENSG00000171791 ENSG00000139112 ENSG00000111335 ENSG00000081041 ENSG00000023445 ENSG00000132357 ENSG00000187796 | CXCL8 GBP3 GBP2 BCL2 GABARAPL1 OAS2 CXCL2 BIRC3 CARD6 CARD9 | hsa:3576 hsa:2635 hsa:2634 hsa:596 hsa:23710 hsa:4939 hsa:2920 hsa:330 hsa:84674 hsa:64170 |
| hsa03410 | Base excision repair | 4\|253 | 51\|9228 | 0.0505205807330913 | 0.485126252090689 | ENSG00000168496 ENSG00000065057 ENSG00000062822 ENSG00000106628 | FEN1 NTHL1 POLD1 POLD2 | hsa:2237 hsa:4913 hsa:5424 hsa:5425 |
| hsa05323 | Rheumatoid arthritis | 5\|253 | 75\|9228 | 0.0545463458157887 | 0.485126252090689 | ENSG00000107562 ENSG00000169429 ENSG00000092969 ENSG00000081041 ENSG00000137462 | CXCL12 CXCL8 TGFB2 CXCL2 TLR2 | hsa:6387 hsa:3576 hsa:7042 hsa:2920 hsa:7097 |
| hsa04950 | Maturity onset diabetes of the young | 2\|253 | 14\|9228 | 0.0548529599473643 | 0.485126252090689 | ENSG00000164749 ENSG00000116833 | HNF4G NR5A2 | hsa:3174 hsa:2494 |
| hsa05166 | Human T-cell leukemia virus 1 infection | 12\|253 | 261\|9228 | 0.0554217030342341 | 0.485126252090689 | novel.507 ENSG00000157613 ENSG00000117399 ENSG00000092969 ENSG00000135476 ENSG00000101412 ENSG00000145386 ENSG00000164742 ENSG00000117394 novel.600 ENSG00000164362 ENSG00000157456 | - CREB3L1 CDC20 TGFB2 ESPL1 E2F1 CCNA2 ADCY1 SLC2A1 - TERT CCNB2 | hsa:1870 hsa:90993 hsa:991 hsa:7042 hsa:9700 hsa:1869 hsa:890 hsa:107 hsa:6513 hsa:1388 hsa:7015 hsa:9133 |
| hsa05145 | Toxoplasmosis | 7\|253 | 125\|9228 | 0.0559390538879936 | 0.485126252090689 | ENSG00000058085 ENSG00000092969 ENSG00000171791 ENSG00000135862 ENSG00000053747 ENSG00000023445 ENSG00000137462 | LAMC2 TGFB2 BCL2 LAMC1 LAMA3 BIRC3 TLR2 | hsa:3918 hsa:7042 hsa:596 hsa:3915 hsa:3909 hsa:330 hsa:7097 |
| hsa04915 | Estrogen signaling pathway | 9\|253 | 180\|9228 | 0.0594010307089812 | 0.485126252090689 | ENSG00000082175 ENSG00000164850 ENSG00000157613 ENSG00000136928 ENSG00000160182 ENSG00000131759 ENSG00000171791 ENSG00000164742 novel.600 | PGR GPER1 CREB3L1 GABBR2 TFF1 RARA BCL2 ADCY1 - | hsa:5241 hsa:2852 hsa:90993 hsa:9568 hsa:7031 hsa:5914 hsa:596 hsa:107 hsa:1388 |
| hsa01523 | Antifolate resistance | 3\|253 | 33\|9228 | 0.0607687788250594 | 0.485126252090689 | ENSG00000125257 ENSG00000176890 ENSG00000173638 | ABCC4 TYMS SLC19A1 | hsa:10257 hsa:7298 hsa:6573 |
| hsa05144 | Malaria | 3\|253 | 33\|9228 | 0.0607687788250594 | 0.485126252090689 | ENSG00000169429 ENSG00000092969 ENSG00000137462 | CXCL8 TGFB2 TLR2 | hsa:3576 hsa:7042 hsa:7097 |
| hsa04727 | GABAergic synapse | 5\|253 | 78\|9228 | 0.0625435786604435 | 0.485126252090689 | ENSG00000136928 ENSG00000127588 ENSG00000187730 ENSG00000139112 ENSG00000164742 | GABBR2 GNG13 GABRD GABARAPL1 ADCY1 | hsa:9568 hsa:51764 hsa:2563 hsa:23710 hsa:107 |
| hsa04911 | Insulin secretion | 5\|253 | 78\|9228 | 0.0625435786604435 | 0.485126252090689 | ENSG00000157613 ENSG00000104783 ENSG00000164742 ENSG00000117394 novel.600 | CREB3L1 KCNN4 ADCY1 SLC2A1 - | hsa:90993 hsa:3783 hsa:107 hsa:6513 hsa:1388 |
| hsa04923 | Regulation of lipolysis in adipocytes | 4\|253 | 55\|9228 | 0.0634395868118593 | 0.485126252090689 | ENSG00000185532 ENSG00000164128 ENSG00000135346 ENSG00000164742 | PRKG1 NPY1R CGA ADCY1 | hsa:5592 hsa:4886 hsa:1081 hsa:107 |
| hsa00410 | beta-Alanine metabolism | 3\|253 | 34\|9228 | 0.0653517439609575 | 0.485470097995684 | ENSG00000259583 ENSG00000184254 ENSG00000137124 | AC015712.2 ALDH1A3 ALDH1B1 | hsa:220 hsa:220 hsa:219 |
| hsa00330 | Arginine and proline metabolism | 4\|253 | 58\|9228 | 0.0741587206482326 | 0.534545668873566 | ENSG00000104524 ENSG00000130066 ENSG00000166165 ENSG00000137124 | PYCR3 SAT1 CKB ALDH1B1 | hsa:65263 hsa:6303 hsa:1152 hsa:219 |
| hsa04725 | Cholinergic synapse | 6\|253 | 108\|9228 | 0.076069960570469 | 0.534545668873566 | ENSG00000157613 ENSG00000127588 ENSG00000168539 ENSG00000171791 ENSG00000164742 ENSG00000087085 | CREB3L1 GNG13 CHRM1 BCL2 ADCY1 ACHE | hsa:90993 hsa:51764 hsa:1128 hsa:596 hsa:107 hsa:43 |
| hsa04061 | Viral protein interaction with cytokine and cytokine receptor | 4\|253 | 61\|9228 | 0.0857330588115478 | 0.570424236603067 | ENSG00000107562 ENSG00000169429 ENSG00000006606 ENSG00000081041 | CXCL12 CXCL8 CCL26 CXCL2 | hsa:6387 hsa:3576 hsa:10344 hsa:2920 |
| hsa00480 | Glutathione metabolism | 4\|253 | 62\|9228 | 0.0897758356475133 | 0.570424236603067 | ENSG00000213366 ENSG00000167741 ENSG00000166825 ENSG00000241720 | GSTM2 GGT6 ANPEP AL158847.1 | hsa:2946 hsa:124975 hsa:290 hsa:2947 |
| hsa00350 | Tyrosine metabolism | 3\|253 | 39\|9228 | 0.090503595353841 | 0.570424236603067 | ENSG00000259583 ENSG00000184254 ENSG00000180176 | AC015712.2 ALDH1A3 TH | hsa:220 hsa:220 hsa:7054 |
| hsa03430 | Mismatch repair | 3\|253 | 39\|9228 | 0.090503595353841 | 0.570424236603067 | ENSG00000049541 ENSG00000062822 ENSG00000106628 | RFC2 POLD1 POLD2 | hsa:5982 hsa:5424 hsa:5425 |
| hsa01524 | Platinum drug resistance | 5\|253 | 88\|9228 | 0.0936856567854724 | 0.570424236603067 | ENSG00000213366 ENSG00000089685 ENSG00000171791 ENSG00000023445 ENSG00000241720 | GSTM2 BIRC5 BCL2 BIRC3 AL158847.1 | hsa:2946 hsa:332 hsa:596 hsa:330 hsa:2947 |
| hsa00360 | Phenylalanine metabolism | 2\|253 | 19\|9228 | 0.0943393929766611 | 0.570424236603067 | ENSG00000259583 ENSG00000184254 | AC015712.2 ALDH1A3 | hsa:220 hsa:220 |
| hsa04114 | Oocyte meiosis | 8\|253 | 171\|9228 | 0.0979927209965503 | 0.579047896797797 | ENSG00000082175 ENSG00000127564 ENSG00000166851 ENSG00000117399 ENSG00000135476 ENSG00000164742 ENSG00000157456 ENSG00000116670 | PGR PKMYT1 PLK1 CDC20 ESPL1 ADCY1 CCNB2 MAD2L2 | hsa:5241 hsa:9088 hsa:5347 hsa:991 hsa:9700 hsa:107 hsa:9133 hsa:10459 |
| hsa04270 | Vascular smooth muscle contraction | 7\|253 | 146\|9228 | 0.106381978384384 | 0.57990360720483 | ENSG00000078401 ENSG00000185532 ENSG00000122679 ENSG00000122786 ENSG00000148926 ENSG00000164742 ENSG00000188257 | EDN1 PRKG1 RAMP3 CALD1 ADM ADCY1 PLA2G2A | hsa:1906 hsa:5592 hsa:10268 hsa:800 hsa:133 hsa:107 hsa:5320 |
| hsa04360 | Axon guidance | 9\|253 | 204\|9228 | 0.108336414161774 | 0.57990360720483 | ENSG00000107562 ENSG00000074527 ENSG00000157851 ENSG00000112902 ENSG00000167680 ENSG00000065320 ENSG00000196220 ENSG00000172602 ENSG00000044524 | CXCL12 NTN4 DPYSL5 SEMA5A SEMA6B NTN1 SRGAP3 RND1 EPHA3 | hsa:6387 hsa:59277 hsa:56896 hsa:9037 hsa:10501 hsa:9423 hsa:9901 hsa:27289 hsa:2042 |
| hsa05142 | Chagas disease (American trypanosomiasis) | 5\|253 | 93\|9228 | 0.111746601280515 | 0.57990360720483 | ENSG00000169429 ENSG00000092969 ENSG00000164742 ENSG00000168398 ENSG00000137462 | CXCL8 TGFB2 ADCY1 BDKRB2 TLR2 | hsa:3576 hsa:7042 hsa:107 hsa:624 hsa:7097 |
| hsa04977 | Vitamin digestion and absorption | 2\|253 | 21\|9228 | 0.11185721893165 | 0.57990360720483 | ENSG00000086205 ENSG00000173638 | FOLH1 SLC19A1 | hsa:2346 hsa:6573 |
| hsa04929 | GnRH secretion | 4\|253 | 68\|9228 | 0.115874266822182 | 0.57990360720483 | ENSG00000164850 ENSG00000135346 ENSG00000136928 ENSG00000104783 | GPER1 CGA GABBR2 KCNN4 | hsa:2852 hsa:1081 hsa:9568 hsa:3783 |
| hsa05321 | Inflammatory bowel disease (IBD) | 3\|253 | 44\|9228 | 0.119023838353315 | 0.57990360720483 | ENSG00000092969 ENSG00000138378 ENSG00000137462 | TGFB2 STAT4 TLR2 | hsa:7042 hsa:6775 hsa:7097 |
| hsa04742 | Taste transduction | 3\|253 | 45\|9228 | 0.12508605279099 | 0.57990360720483 | ENSG00000136928 ENSG00000127588 ENSG00000164626 | GABBR2 GNG13 KCNK5 | hsa:9568 hsa:51764 hsa:8645 |
| hsa05032 | Morphine addiction | 4\|253 | 70\|9228 | 0.125237628539926 | 0.57990360720483 | ENSG00000136928 ENSG00000127588 ENSG00000187730 ENSG00000164742 | GABBR2 GNG13 GABRD ADCY1 | hsa:9568 hsa:51764 hsa:2563 hsa:107 |
| hsa04610 | Complement and coagulation cascades | 4\|253 | 71\|9228 | 0.130036126096799 | 0.57990360720483 | ENSG00000237248 ENSG00000175899 ENSG00000168398 ENSG00000131187 | LINC00987 A2M BDKRB2 F12 | hsa:2 hsa:2 hsa:624 hsa:2161 |
| hsa04215 | Apoptosis - multiple species | 3\|253 | 46\|9228 | 0.131257515404487 | 0.57990360720483 | ENSG00000089685 ENSG00000171791 ENSG00000023445 | BIRC5 BCL2 BIRC3 | hsa:332 hsa:596 hsa:330 |
| hsa04064 | NF-kappa B signaling pathway | 5\|253 | 98\|9228 | 0.131355340877762 | 0.57990360720483 | ENSG00000107562 ENSG00000169429 ENSG00000171791 ENSG00000081041 ENSG00000023445 | CXCL12 CXCL8 BCL2 CXCL2 BIRC3 | hsa:6387 hsa:3576 hsa:596 hsa:2920 hsa:330 |
| hsa04024 | cAMP signaling pathway | 9\|253 | 214\|9228 | 0.133910845135558 | 0.57990360720483 | ENSG00000125257 ENSG00000078401 ENSG00000157613 ENSG00000164128 ENSG00000135346 ENSG00000136928 ENSG00000168539 ENSG00000125398 ENSG00000164742 | ABCC4 EDN1 CREB3L1 NPY1R CGA GABBR2 CHRM1 SOX9 ADCY1 | hsa:10257 hsa:1906 hsa:90993 hsa:4886 hsa:1081 hsa:9568 hsa:1128 hsa:6662 hsa:107 |
| hsa05204 | Chemical carcinogenesis | 4\|253 | 72\|9228 | 0.134910083263608 | 0.57990360720483 | ENSG00000213366 ENSG00000259583 ENSG00000184254 ENSG00000241720 | GSTM2 AC015712.2 ALDH1A3 AL158847.1 | hsa:2946 hsa:220 hsa:220 hsa:2947 |
| hsa01522 | Endocrine resistance | 5\|253 | 99\|9228 | 0.135453351364967 | 0.57990360720483 | ENSG00000164850 novel.507 ENSG00000101412 ENSG00000171791 ENSG00000164742 | GPER1 - E2F1 BCL2 ADCY1 | hsa:2852 hsa:1870 hsa:1869 hsa:596 hsa:107 |
| hsa05030 | Cocaine addiction | 3\|253 | 47\|9228 | 0.137533829702552 | 0.57990360720483 | ENSG00000157613 ENSG00000180176 novel.600 | CREB3L1 TH - | hsa:90993 hsa:7054 hsa:1388 |
| hsa04514 | Cell adhesion molecules (CAMs) | 6\|253 | 128\|9228 | 0.139438976763708 | 0.57990360720483 | ENSG00000179776 ENSG00000173482 ENSG00000134258 ENSG00000107738 ENSG00000188404 ENSG00000105695 | CDH5 PTPRM VTCN1 VSIR SELL MAG | hsa:1003 hsa:5797 hsa:79679 hsa:64115 hsa:6402 hsa:4099 |
| hsa04918 | Thyroid hormone synthesis | 4\|253 | 73\|9228 | 0.139857639988752 | 0.57990360720483 | ENSG00000157613 ENSG00000135346 ENSG00000164742 novel.600 | CREB3L1 CGA ADCY1 - | hsa:90993 hsa:1081 hsa:107 hsa:1388 |
| hsa04976 | Bile secretion | 4\|253 | 73\|9228 | 0.139857639988752 | 0.57990360720483 | ENSG00000125257 ENSG00000080493 ENSG00000164742 ENSG00000117394 | ABCC4 SLC4A4 ADCY1 SLC2A1 | hsa:10257 hsa:8671 hsa:107 hsa:6513 |
| hsa03440 | Homologous recombination | 3\|253 | 48\|9228 | 0.143910590551196 | 0.57990360720483 | ENSG00000154920 ENSG00000062822 ENSG00000106628 | EME1 POLD1 POLD2 | hsa:146956 hsa:5424 hsa:5425 |
| hsa05219 | Bladder cancer | 3\|253 | 48\|9228 | 0.143910590551196 | 0.57990360720483 | novel.507 ENSG00000169429 ENSG00000101412 | - CXCL8 E2F1 | hsa:1870 hsa:3576 hsa:1869 |
| hsa05418 | Fluid shear stress and atherosclerosis | 8\|253 | 188\|9228 | 0.144975901801208 | 0.57990360720483 | ENSG00000179776 ENSG00000078401 ENSG00000005961 ENSG00000213366 ENSG00000128052 ENSG00000116701 ENSG00000171791 ENSG00000241720 | CDH5 EDN1 ITGA2B GSTM2 KDR NCF2 BCL2 AL158847.1 | hsa:1003 hsa:1906 hsa:3674 hsa:2946 hsa:3791 hsa:4688 hsa:596 hsa:2947 |
| hsa04211 | Longevity regulating pathway | 5\|253 | 103\|9228 | 0.152400546039166 | 0.596618168116342 | ENSG00000157613 ENSG00000109819 ENSG00000164742 novel.600 ENSG00000132170 | CREB3L1 PPARGC1A ADCY1 - PPARG | hsa:90993 hsa:10891 hsa:107 hsa:1388 hsa:5468 |
| hsa05165 | Human papillomavirus infection | 14\|253 | 377\|9228 | 0.153743912553057 | 0.596618168116342 | ENSG00000135547 ENSG00000005961 ENSG00000157613 ENSG00000058085 ENSG00000188153 ENSG00000169031 ENSG00000101412 ENSG00000145386 ENSG00000131462 ENSG00000135862 ENSG00000109062 ENSG00000053747 ENSG00000115221 ENSG00000164362 | HEY2 ITGA2B CREB3L1 LAMC2 COL4A5 COL4A3 E2F1 CCNA2 TUBG1 LAMC1 SLC9A3R1 LAMA3 ITGB6 TERT | hsa:23493 hsa:3674 hsa:90993 hsa:3918 hsa:1287 hsa:1285 hsa:1869 hsa:890 hsa:7283 hsa:3915 hsa:9368 hsa:3909 hsa:3694 hsa:7015 |
| hsa04540 | Gap junction | 5\|253 | 104\|9228 | 0.15677092617179 | 0.599418247127432 | ENSG00000185532 ENSG00000214222 ENSG00000123416 ENSG00000164742 ENSG00000104833 | PRKG1 TUBBP2 TUBA1B ADCY1 TUBB4A | hsa:5592 hsa:203068 hsa:10376 hsa:107 hsa:10382 |
| hsa02010 | ABC transporters | 3\|253 | 51\|9228 | 0.163599540542997 | 0.616462036828685 | ENSG00000125257 ENSG00000198691 ENSG00000144452 | ABCC4 ABCA4 ABCA12 | hsa:10257 hsa:24 hsa:26154 |
| hsa00600 | Sphingolipid metabolism | 3\|253 | 52\|9228 | 0.170334140180221 | 0.632669663526534 | ENSG00000177076 ENSG00000168350 ENSG00000102393 | ACER2 DEGS2 GLA | hsa:340485 hsa:123099 hsa:2717 |
| hsa00100 | Steroid biosynthesis | 2\|253 | 29\|9228 | 0.188248365367176 | 0.689360211203742 | ENSG00000167780 ENSG00000147155 | SOAT2 EBP | hsa:8435 hsa:10682 |
| hsa03420 | Nucleotide excision repair | 3\|253 | 56\|9228 | 0.198015313203954 | 0.715055297680945 | ENSG00000049541 ENSG00000062822 ENSG00000106628 | RFC2 POLD1 POLD2 | hsa:5982 hsa:5424 hsa:5425 |
| hsa05202 | Transcriptional misregulation in cancer | 8\|253 | 205\|9228 | 0.201111868226034 | 0.716288845736558 | ENSG00000164120 ENSG00000169429 ENSG00000146674 ENSG00000131759 ENSG00000145386 ENSG00000023445 ENSG00000058091 ENSG00000132170 | HPGD CXCL8 IGFBP3 RARA CCNA2 BIRC3 CDK14 PPARG | hsa:3248 hsa:3576 hsa:3486 hsa:5914 hsa:890 hsa:330 hsa:5218 hsa:5468 |
| hsa00983 | Drug metabolism - other enzymes | 4\|253 | 91\|9228 | 0.239237597075415 | 0.83064131920038 | ENSG00000213366 ENSG00000167900 ENSG00000128951 ENSG00000241720 | GSTM2 TK1 DUT AL158847.1 | hsa:2946 hsa:7083 hsa:1854 hsa:2947 |
| hsa00430 | Taurine and hypotaurine metabolism | 1\|253 | 10\|9228 | 0.24280284715088 | 0.83064131920038 | ENSG00000167741 | GGT6 | hsa:124975 |
| hsa00920 | Sulfur metabolism | 1\|253 | 10\|9228 | 0.24280284715088 | 0.83064131920038 | ENSG00000143416 | SELENBP1 | hsa:8991 |
| hsa00270 | Cysteine and methionine metabolism | 3\|253 | 63\|9228 | 0.248675535583751 | 0.839683626646432 | ENSG00000151224 ENSG00000088305 ENSG00000092621 | MAT1A DNMT3B PHGDH | hsa:4143 hsa:1789 hsa:26227 |
| hsa04713 | Circadian entrainment | 4\|253 | 94\|9228 | 0.257229644300325 | 0.846742590798202 | ENSG00000185532 ENSG00000108551 ENSG00000127588 ENSG00000164742 | PRKG1 RASD1 GNG13 ADCY1 | hsa:5592 hsa:51655 hsa:51764 hsa:107 |
| hsa04927 | Cortisol synthesis and secretion | 3\|253 | 65\|9228 | 0.263513585527816 | 0.846742590798202 | ENSG00000157613 ENSG00000164742 novel.600 | CREB3L1 ADCY1 - | hsa:90993 hsa:107 hsa:1388 |
| hsa05031 | Amphetamine addiction | 3\|253 | 65\|9228 | 0.263513585527816 | 0.846742590798202 | ENSG00000157613 ENSG00000180176 novel.600 | CREB3L1 TH - | hsa:90993 hsa:7054 hsa:1388 |
| hsa04668 | TNF signaling pathway | 5\|253 | 126\|9228 | 0.263792884056363 | 0.846742590798202 | ENSG00000078401 ENSG00000157613 ENSG00000081041 novel.600 ENSG00000023445 | EDN1 CREB3L1 CXCL2 - BIRC3 | hsa:1906 hsa:90993 hsa:2920 hsa:1388 hsa:330 |
| hsa00561 | Glycerolipid metabolism | 3\|253 | 67\|9228 | 0.278458650744309 | 0.88291767309171 | ENSG00000101670 ENSG00000137124 ENSG00000102393 | LIPG ALDH1B1 GLA | hsa:9388 hsa:219 hsa:2717 |
| hsa00072 | Synthesis and degradation of ketone bodies | 1\|253 | 12\|9228 | 0.283799165379048 | 0.8890094337175 | ENSG00000134240 | HMGCS2 | hsa:3158 |
| hsa04216 | Ferroptosis | 3\|253 | 69\|9228 | 0.293482824078866 | 0.895916584989771 | ENSG00000047457 ENSG00000130066 ENSG00000240216 | CP SAT1 CPHL1P | hsa:1356 hsa:6303 hsa:1356 |
| hsa04071 | Sphingolipid signaling pathway | 5\|253 | 132\|9228 | 0.295584301275058 | 0.895916584989771 | ENSG00000177076 ENSG00000168350 ENSG00000171791 ENSG00000168398 ENSG00000180739 | ACER2 DEGS2 BCL2 BDKRB2 S1PR5 | hsa:340485 hsa:123099 hsa:596 hsa:624 hsa:53637 |
| hsa05169 | Epstein-Barr virus infection | 8\|253 | 230\|9228 | 0.296341639650463 | 0.895916584989771 | ENSG00000026025 novel.507 ENSG00000168032 ENSG00000101412 ENSG00000171791 ENSG00000145386 ENSG00000111335 ENSG00000137462 | VIM - ENTPD3 E2F1 BCL2 CCNA2 OAS2 TLR2 | hsa:7431 hsa:1870 hsa:956 hsa:1869 hsa:596 hsa:890 hsa:4939 hsa:7097 |
| hsa04934 | Cushing syndrome | 6\|253 | 166\|9228 | 0.303980453840345 | 0.908447333315972 | novel.507 ENSG00000157613 ENSG00000108551 ENSG00000101412 ENSG00000164742 novel.600 | - CREB3L1 RASD1 E2F1 ADCY1 - | hsa:1870 hsa:90993 hsa:51655 hsa:1869 hsa:107 hsa:1388 |
| hsa00010 | Glycolysis / Gluconeogenesis | 4\|253 | 104\|9228 | 0.31879162424191 | 0.915686554977935 | ENSG00000259583 ENSG00000184254 ENSG00000137124 ENSG00000186197 | AC015712.2 ALDH1A3 ALDH1B1 EDARADD | hsa:220 hsa:220 hsa:219 hsa:2023 |
| hsa00860 | Porphyrin and chlorophyll metabolism | 2\|253 | 43\|9228 | 0.330755624899464 | 0.915686554977935 | ENSG00000047457 ENSG00000240216 | CP CPHL1P | hsa:1356 hsa:1356 |
| hsa05410 | Hypertrophic cardiomyopathy (HCM) | 4\|253 | 106\|9228 | 0.331282926851914 | 0.915686554977935 | ENSG00000078401 ENSG00000005961 ENSG00000092969 ENSG00000115221 | EDN1 ITGA2B TGFB2 ITGB6 | hsa:1906 hsa:3674 hsa:7042 hsa:3694 |
| hsa05034 | Alcoholism | 7\|253 | 205\|9228 | 0.331375954742587 | 0.915686554977935 | ENSG00000157613 ENSG00000127588 ENSG00000184260 ENSG00000124635 ENSG00000180176 novel.600 ENSG00000218690 | CREB3L1 GNG13 HIST2H2AC HIST1H2BJ TH - HIST1H2APS4 | hsa:90993 hsa:51764 hsa:8338 hsa:8970 hsa:7054 hsa:1388 hsa:8336 |
| hsa04015 | Rap1 signaling pathway | 8\|253 | 240\|9228 | 0.337204148858878 | 0.915686554977935 | ENSG00000005961 ENSG00000160867 ENSG00000152689 ENSG00000128052 ENSG00000164742 ENSG00000082074 ENSG00000037280 ENSG00000233328 | ITGA2B FGFR4 RASGRP3 KDR ADCY1 FYB1 FLT4 PFN1P1 | hsa:3674 hsa:2264 hsa:25780 hsa:3791 hsa:107 hsa:2533 hsa:2324 hsa:5216 |
| hsa04022 | cGMP-PKG signaling pathway | 7\|253 | 207\|9228 | 0.340298946783167 | 0.915686554977935 | ENSG00000185532 ENSG00000157613 ENSG00000116741 ENSG00000164742 ENSG00000168398 novel.600 ENSG00000121361 | PRKG1 CREB3L1 RGS2 ADCY1 BDKRB2 - KCNJ8 | hsa:5592 hsa:90993 hsa:5997 hsa:107 hsa:624 hsa:1388 hsa:3764 |
| hsa05210 | Colorectal cancer | 4\|253 | 108\|9228 | 0.343800118955873 | 0.915686554977935 | ENSG00000092969 ENSG00000109321 ENSG00000089685 ENSG00000171791 | TGFB2 AREG BIRC5 BCL2 | hsa:7042 hsa:374 hsa:332 hsa:596 |
| hsa04060 | Cytokine-cytokine receptor interaction | 7\|253 | 208\|9228 | 0.34477260240847 | 0.915686554977935 | ENSG00000107562 ENSG00000181634 ENSG00000169429 ENSG00000092969 ENSG00000006606 ENSG00000081041 ENSG00000104998 | CXCL12 TNFSF15 CXCL8 TGFB2 CCL26 CXCL2 IL27RA | hsa:6387 hsa:9966 hsa:3576 hsa:7042 hsa:10344 hsa:2920 hsa:9466 |
| hsa04062 | Chemokine signaling pathway | 6\|253 | 175\|9228 | 0.347723977275585 | 0.915686554977935 | ENSG00000107562 ENSG00000169429 ENSG00000127588 ENSG00000164742 ENSG00000006606 ENSG00000081041 | CXCL12 CXCL8 GNG13 ADCY1 CCL26 CXCL2 | hsa:6387 hsa:3576 hsa:51764 hsa:107 hsa:10344 hsa:2920 |
| hsa04917 | Prolactin signaling pathway | 3\|253 | 77\|9228 | 0.353856029298908 | 0.915686554977935 | ENSG00000135346 ENSG00000114737 ENSG00000180176 | CGA CISH TH | hsa:1081 hsa:1154 hsa:7054 |
| hsa00603 | Glycosphingolipid biosynthesis - globo and isoglobo series | 1\|253 | 16\|9228 | 0.359276635694307 | 0.915686554977935 | ENSG00000102393 | GLA | hsa:2717 |
| hsa04970 | Salivary secretion | 3\|253 | 78\|9228 | 0.36138498436027 | 0.915686554977935 | ENSG00000185532 ENSG00000104783 ENSG00000164742 | PRKG1 KCNN4 ADCY1 | hsa:5592 hsa:3783 hsa:107 |
| hsa05414 | Dilated cardiomyopathy (DCM) | 4\|253 | 112\|9228 | 0.368858144301464 | 0.915686554977935 | ENSG00000005961 ENSG00000092969 ENSG00000164742 ENSG00000115221 | ITGA2B TGFB2 ADCY1 ITGB6 | hsa:3674 hsa:7042 hsa:107 hsa:3694 |
| hsa05140 | Leishmaniasis | 3\|253 | 79\|9228 | 0.368900947264493 | 0.915686554977935 | ENSG00000092969 ENSG00000116701 ENSG00000137462 | TGFB2 NCF2 TLR2 | hsa:7042 hsa:4688 hsa:7097 |
| hsa04962 | Vasopressin-regulated water reabsorption | 2\|253 | 47\|9228 | 0.370781006401356 | 0.915686554977935 | ENSG00000157613 ENSG00000111348 | CREB3L1 ARHGDIB | hsa:90993 hsa:397 |
| hsa04979 | Cholesterol metabolism | 2\|253 | 47\|9228 | 0.370781006401356 | 0.915686554977935 | ENSG00000101670 ENSG00000167780 | LIPG SOAT2 | hsa:9388 hsa:8435 |
| hsa00604 | Glycosphingolipid biosynthesis - ganglio series | 1\|253 | 17\|9228 | 0.376873575682295 | 0.915686554977935 | ENSG00000135454 | B4GALNT1 | hsa:2583 |
| hsa03450 | Non-homologous end-joining | 1\|253 | 17\|9228 | 0.376873575682295 | 0.915686554977935 | ENSG00000168496 | FEN1 | hsa:2237 |
| hsa05224 | Breast cancer | 5\|253 | 147\|9228 | 0.377140260471596 | 0.915686554977935 | ENSG00000082175 ENSG00000135547 novel.507 ENSG00000101412 ENSG00000037280 | PGR HEY2 - E2F1 FLT4 | hsa:5241 hsa:23493 hsa:1870 hsa:1869 hsa:2324 |
| hsa05226 | Gastric cancer | 5\|253 | 148\|9228 | 0.382614296824029 | 0.915686554977935 | novel.507 ENSG00000092969 ENSG00000101412 ENSG00000171791 ENSG00000164362 | - TGFB2 E2F1 BCL2 TERT | hsa:1870 hsa:7042 hsa:1869 hsa:596 hsa:7015 |
| hsa05163 | Human cytomegalovirus infection | 8\|253 | 251\|9228 | 0.383072350096086 | 0.915686554977935 | ENSG00000107562 novel.507 ENSG00000157613 ENSG00000169429 ENSG00000127588 ENSG00000101412 ENSG00000164742 novel.600 | CXCL12 - CREB3L1 CXCL8 GNG13 E2F1 ADCY1 - | hsa:6387 hsa:1870 hsa:90993 hsa:3576 hsa:51764 hsa:1869 hsa:107 hsa:1388 |
| hsa03320 | PPAR signaling pathway | 3\|253 | 81\|9228 | 0.383883978817673 | 0.915686554977935 | ENSG00000214456 ENSG00000134240 ENSG00000132170 | PLIN5 HMGCS2 PPARG | hsa:440503 hsa:3158 hsa:5468 |
| hsa05215 | Prostate cancer | 4\|253 | 115\|9228 | 0.387621687006631 | 0.916196714742947 | novel.507 ENSG00000157613 ENSG00000101412 ENSG00000171791 | - CREB3L1 E2F1 BCL2 | hsa:1870 hsa:90993 hsa:1869 hsa:596 |
| hsa05033 | Nicotine addiction | 1\|253 | 18\|9228 | 0.39398908815134 | 0.919609076442582 | ENSG00000187730 | GABRD | hsa:2563 |
| hsa05167 | Kaposi sarcoma-associated herpesvirus infection | 7\|253 | 220\|9228 | 0.398841416310499 | 0.919609076442582 | novel.507 ENSG00000169429 ENSG00000127588 ENSG00000159200 ENSG00000101412 ENSG00000139112 ENSG00000081041 | - CXCL8 GNG13 RCAN1 E2F1 GABARAPL1 CXCL2 | hsa:1870 hsa:3576 hsa:51764 hsa:1827 hsa:1869 hsa:23710 hsa:2920 |
| hsa01230 | Biosynthesis of amino acids | 4\|253 | 118\|9228 | 0.406314008829185 | 0.919609076442582 | ENSG00000104524 ENSG00000186197 ENSG00000151224 ENSG00000092621 | PYCR3 EDARADD MAT1A PHGDH | hsa:65263 hsa:2023 hsa:4143 hsa:26227 |
| hsa04913 | Ovarian steroidogenesis | 2\|253 | 51\|9228 | 0.40982830895255 | 0.919609076442582 | ENSG00000135346 ENSG00000164742 | CGA ADCY1 | hsa:1081 hsa:107 |
| hsa04614 | Renin-angiotensin system | 1\|253 | 19\|9228 | 0.410636293438823 | 0.919609076442582 | ENSG00000166825 | ANPEP | hsa:290 |
| hsa00310 | Lysine degradation | 3\|253 | 85\|9228 | 0.413589410156625 | 0.919609076442582 | ENSG00000008311 ENSG00000101945 ENSG00000137124 | AASS SUV39H1 ALDH1B1 | hsa:10157 hsa:6839 hsa:219 |
| hsa04120 | Ubiquitin mediated proteolysis | 7\|253 | 224\|9228 | 0.416910776845441 | 0.919609076442582 | ENSG00000224126 ENSG00000117399 ENSG00000108106 ENSG00000114423 ENSG00000101871 ENSG00000023445 ENSG00000224080 | UBE2SP2 CDC20 UBE2S CBLB MID1 BIRC3 UBE2FP1 | hsa:27338 hsa:991 hsa:27338 hsa:868 hsa:4281 hsa:330 hsa:140739 |
| hsa00910 | Nitrogen metabolism | 1\|253 | 20\|9228 | 0.426827955699652 | 0.919609076442582 | ENSG00000074410 | CA12 | hsa:771 |
| hsa04935 | Growth hormone synthesis, secretion and action | 4\|253 | 122\|9228 | 0.431060586767249 | 0.919609076442582 | ENSG00000157613 ENSG00000146674 ENSG00000164742 novel.600 | CREB3L1 IGFBP3 ADCY1 - | hsa:90993 hsa:3486 hsa:107 hsa:1388 |
| hsa04145 | Phagosome | 6\|253 | 192\|9228 | 0.431323776344981 | 0.919609076442582 | ENSG00000214222 ENSG00000123416 ENSG00000116701 ENSG00000102879 ENSG00000137462 ENSG00000104833 | TUBBP2 TUBA1B NCF2 CORO1A TLR2 TUBB4A | hsa:203068 hsa:10376 hsa:4688 hsa:11151 hsa:7097 hsa:10382 |
| hsa04922 | Glucagon signaling pathway | 4\|253 | 123\|9228 | 0.437206901120025 | 0.919609076442582 | ENSG00000157613 ENSG00000109819 ENSG00000275993 ENSG00000117394 | CREB3L1 PPARGC1A SIK1B SLC2A1 | hsa:90993 hsa:10891 hsa:102724428 hsa:6513 |
| hsa00260 | Glycine, serine and threonine metabolism | 2\|253 | 54\|9228 | 0.438326720567751 | 0.919609076442582 | ENSG00000123453 ENSG00000092621 | SARDH PHGDH | hsa:1757 hsa:26227 |
| hsa00053 | Ascorbate and aldarate metabolism | 1\|253 | 21\|9228 | 0.442576492538052 | 0.919609076442582 | ENSG00000137124 | ALDH1B1 | hsa:219 |
| hsa05211 | Renal cell carcinoma | 3\|253 | 90\|9228 | 0.450059677398356 | 0.919609076442582 | ENSG00000116016 ENSG00000092969 ENSG00000117394 | EPAS1 TGFB2 SLC2A1 | hsa:2034 hsa:7042 hsa:6513 |
| hsa04371 | Apelin signaling pathway | 5\|253 | 161\|9228 | 0.453269065270296 | 0.919609076442582 | ENSG00000127588 ENSG00000118523 ENSG00000109819 ENSG00000139112 ENSG00000164742 | GNG13 CTGF PPARGC1A GABARAPL1 ADCY1 | hsa:51764 hsa:1490 hsa:10891 hsa:23710 hsa:107 |
| hsa04961 | Endocrine and other factor-regulated calcium reabsorption | 2\|253 | 56\|9228 | 0.456906204613943 | 0.919609076442582 | ENSG00000197959 ENSG00000168398 | DNM3 BDKRB2 | hsa:26052 hsa:624 |
| hsa04012 | ErbB signaling pathway | 3\|253 | 94\|9228 | 0.478564692483762 | 0.919609076442582 | ENSG00000109321 ENSG00000174808 ENSG00000114423 | AREG BTC CBLB | hsa:374 hsa:685 hsa:868 |
| hsa04657 | IL-17 signaling pathway | 3\|253 | 94\|9228 | 0.478564692483762 | 0.919609076442582 | ENSG00000169429 ENSG00000137745 ENSG00000081041 | CXCL8 MMP13 CXCL2 | hsa:3576 hsa:4322 hsa:2920 |
| hsa04972 | Pancreatic secretion | 3\|253 | 94\|9228 | 0.478564692483762 | 0.919609076442582 | ENSG00000080493 ENSG00000164742 ENSG00000188257 | SLC4A4 ADCY1 PLA2G2A | hsa:8671 hsa:107 hsa:5320 |
| hsa00280 | Valine, leucine and isoleucine degradation | 2\|253 | 59\|9228 | 0.484099377695008 | 0.919609076442582 | ENSG00000134240 ENSG00000137124 | HMGCS2 ALDH1B1 | hsa:3158 hsa:219 |
| hsa05212 | Pancreatic cancer | 3\|253 | 96\|9228 | 0.492559175630896 | 0.919609076442582 | novel.507 ENSG00000092969 ENSG00000101412 | - TGFB2 E2F1 | hsa:1870 hsa:7042 hsa:1869 |
| hsa05225 | Hepatocellular carcinoma | 6\|253 | 205\|9228 | 0.494200099992938 | 0.919609076442582 | novel.507 ENSG00000213366 ENSG00000092969 ENSG00000101412 ENSG00000164362 ENSG00000241720 | - GSTM2 TGFB2 E2F1 TERT AL158847.1 | hsa:1870 hsa:2946 hsa:7042 hsa:1869 hsa:7015 hsa:2947 |
| hsa04728 | Dopaminergic synapse | 4\|253 | 133\|9228 | 0.497458798491271 | 0.919609076442582 | ENSG00000157613 ENSG00000127588 ENSG00000180176 novel.600 | CREB3L1 GNG13 TH - | hsa:90993 hsa:51764 hsa:7054 hsa:1388 |
| hsa04152 | AMPK signaling pathway | 4\|253 | 134\|9228 | 0.503339723012456 | 0.919609076442582 | ENSG00000157613 ENSG00000109819 ENSG00000145386 ENSG00000132170 | CREB3L1 PPARGC1A CCNA2 PPARG | hsa:90993 hsa:10891 hsa:890 hsa:5468 |
| hsa00534 | Glycosaminoglycan biosynthesis - heparan sulfate / heparin | 1\|253 | 26\|9228 | 0.515083812941858 | 0.919609076442582 | ENSG00000002587 | HS3ST1 | hsa:9957 |
| hsa05320 | Autoimmune thyroid disease | 1\|253 | 26\|9228 | 0.515083812941858 | 0.919609076442582 | ENSG00000135346 | CGA | hsa:1081 |
| hsa05220 | Chronic myeloid leukemia | 3\|253 | 100\|9228 | 0.51997955410553 | 0.919609076442582 | novel.507 ENSG00000092969 ENSG00000101412 | - TGFB2 E2F1 | hsa:1870 hsa:7042 hsa:1869 |
| hsa00592 | alpha-Linolenic acid metabolism | 1\|253 | 27\|9228 | 0.528416109760561 | 0.919609076442582 | ENSG00000188257 | PLA2G2A | hsa:5320 |
| hsa04916 | Melanogenesis | 3\|253 | 102\|9228 | 0.53338737332881 | 0.919609076442582 | ENSG00000078401 ENSG00000157613 ENSG00000164742 | EDN1 CREB3L1 ADCY1 | hsa:1906 hsa:90993 hsa:107 |
| hsa04925 | Aldosterone synthesis and secretion | 3\|253 | 103\|9228 | 0.540012192760728 | 0.919609076442582 | ENSG00000157613 ENSG00000164742 novel.600 | CREB3L1 ADCY1 - | hsa:90993 hsa:107 hsa:1388 |
| hsa00670 | One carbon pool by folate | 1\|253 | 28\|9228 | 0.541383257269591 | 0.919609076442582 | ENSG00000176890 | TYMS | hsa:7298 |
| hsa00790 | Folate biosynthesis | 1\|253 | 28\|9228 | 0.541383257269591 | 0.919609076442582 | ENSG00000180176 | TH | hsa:7054 |
| hsa04964 | Proximal tubule bicarbonate reclamation | 1\|253 | 28\|9228 | 0.541383257269591 | 0.919609076442582 | ENSG00000080493 | SLC4A4 | hsa:8671 |
| hsa05152 | Tuberculosis | 5\|253 | 178\|9228 | 0.541942756948401 | 0.919609076442582 | ENSG00000092969 ENSG00000171791 ENSG00000102879 ENSG00000137462 ENSG00000187796 | TGFB2 BCL2 CORO1A TLR2 CARD9 | hsa:7042 hsa:596 hsa:11151 hsa:7097 hsa:64170 |
| hsa04611 | Platelet activation | 4\|253 | 141\|9228 | 0.543619608445488 | 0.919609076442582 | ENSG00000185532 ENSG00000005961 ENSG00000164742 ENSG00000168542 | PRKG1 ITGA2B ADCY1 COL3A1 | hsa:5592 hsa:3674 hsa:107 hsa:1281 |
| hsa04640 | Hematopoietic cell lineage | 2\|253 | 67\|9228 | 0.552335857200442 | 0.919609076442582 | ENSG00000005961 ENSG00000166825 | ITGA2B ANPEP | hsa:3674 hsa:290 |
| hsa03018 | RNA degradation | 3\|253 | 105\|9228 | 0.553099275151824 | 0.919609076442582 | ENSG00000159388 ENSG00000130520 ENSG00000186197 | BTG2 LSM4 EDARADD | hsa:7832 hsa:25804 hsa:2023 |
| hsa00052 | Galactose metabolism | 1\|253 | 29\|9228 | 0.553995217694677 | 0.919609076442582 | ENSG00000102393 | GLA | hsa:2717 |
| hsa04672 | Intestinal immune network for IgA production | 1\|253 | 29\|9228 | 0.553995217694677 | 0.919609076442582 | ENSG00000107562 | CXCL12 | hsa:6387 |
| hsa04724 | Glutamatergic synapse | 3\|253 | 106\|9228 | 0.559559945490656 | 0.919609076442582 | ENSG00000127588 ENSG00000164742 ENSG00000164418 | GNG13 ADCY1 GRIK2 | hsa:51764 hsa:107 hsa:2898 |
| hsa05162 | Measles | 4\|253 | 144\|9228 | 0.560366217552053 | 0.919609076442582 | ENSG00000171791 ENSG00000114423 ENSG00000111335 ENSG00000137462 | BCL2 CBLB OAS2 TLR2 | hsa:596 hsa:868 hsa:4939 hsa:7097 |
| hsa04810 | Regulation of actin cytoskeleton | 7\|253 | 257\|9228 | 0.561447247908817 | 0.919609076442582 | ENSG00000107562 ENSG00000005961 ENSG00000160867 ENSG00000168539 ENSG00000168398 ENSG00000115221 ENSG00000233328 | CXCL12 ITGA2B FGFR4 CHRM1 BDKRB2 ITGB6 PFN1P1 | hsa:6387 hsa:3674 hsa:2264 hsa:1128 hsa:624 hsa:3694 hsa:5216 |
| hsa00564 | Glycerophospholipid metabolism | 3\|253 | 107\|9228 | 0.565964391555209 | 0.919609076442582 | ENSG00000143845 ENSG00000188257 ENSG00000087085 | ETNK2 PLA2G2A ACHE | hsa:55224 hsa:5320 hsa:43 |
| hsa05218 | Melanoma | 2\|253 | 70\|9228 | 0.576239199265705 | 0.919609076442582 | novel.507 ENSG00000101412 | - E2F1 | hsa:1870 hsa:1869 |
| hsa04210 | Apoptosis | 5\|253 | 185\|9228 | 0.57649520556516 | 0.919609076442582 | ENSG00000162909 ENSG00000123416 ENSG00000089685 ENSG00000171791 ENSG00000023445 | CAPN2 TUBA1B BIRC5 BCL2 BIRC3 | hsa:824 hsa:10376 hsa:332 hsa:596 hsa:330 |
| hsa04217 | Necroptosis | 6\|253 | 223\|9228 | 0.577002999806222 | 0.919609076442582 | ENSG00000162909 ENSG00000138378 ENSG00000184260 ENSG00000171791 ENSG00000023445 ENSG00000218690 | CAPN2 STAT4 HIST2H2AC BCL2 BIRC3 HIST1H2APS4 | hsa:824 hsa:6775 hsa:8338 hsa:596 hsa:330 hsa:8336 |
| hsa00500 | Starch and sucrose metabolism | 1\|253 | 31\|9228 | 0.578192079814811 | 0.919609076442582 | ENSG00000165434 | PGM2L1 | hsa:283209 |
| hsa00591 | Linoleic acid metabolism | 1\|253 | 31\|9228 | 0.578192079814811 | 0.919609076442582 | ENSG00000188257 | PLA2G2A | hsa:5320 |
| hsa00900 | Terpenoid backbone biosynthesis | 1\|253 | 31\|9228 | 0.578192079814811 | 0.919609076442582 | ENSG00000134240 | HMGCS2 | hsa:3158 |
| hsa04630 | JAK-STAT signaling pathway | 4\|253 | 148\|9228 | 0.582171134775095 | 0.919609076442582 | ENSG00000114737 ENSG00000138378 ENSG00000171791 ENSG00000104998 | CISH STAT4 BCL2 IL27RA | hsa:1154 hsa:6775 hsa:596 hsa:9466 |
| hsa00590 | Arachidonic acid metabolism | 2\|253 | 71\|9228 | 0.583998033180575 | 0.919609076442582 | ENSG00000256612 ENSG00000188257 | CYP2B7P PLA2G2A | hsa:1555 hsa:5320 |
| hsa04920 | Adipocytokine signaling pathway | 2\|253 | 71\|9228 | 0.583998033180575 | 0.919609076442582 | ENSG00000109819 ENSG00000117394 | PPARGC1A SLC2A1 | hsa:10891 hsa:6513 |
| hsa04625 | C-type lectin receptor signaling pathway | 3\|253 | 111\|9228 | 0.591007219615574 | 0.919609076442582 | ENSG00000179388 ENSG00000114423 ENSG00000187796 | EGR3 CBLB CARD9 | hsa:1960 hsa:868 hsa:64170 |
| hsa05221 | Acute myeloid leukemia | 2\|253 | 72\|9228 | 0.591651994106726 | 0.919609076442582 | ENSG00000131759 ENSG00000145386 | RARA CCNA2 | hsa:5914 hsa:890 |
| hsa00650 | Butanoate metabolism | 1\|253 | 33\|9228 | 0.601081109634144 | 0.919609076442582 | ENSG00000134240 | HMGCS2 | hsa:3158 |
| hsa01040 | Biosynthesis of unsaturated fatty acids | 1\|253 | 33\|9228 | 0.601081109634144 | 0.919609076442582 | ENSG00000097021 | ACOT7 | hsa:11332 |
| hsa04066 | HIF-1 signaling pathway | 4\|253 | 152\|9228 | 0.603346546187085 | 0.919609076442582 | ENSG00000078401 ENSG00000171791 ENSG00000117394 ENSG00000186197 | EDN1 BCL2 SLC2A1 EDARADD | hsa:1906 hsa:596 hsa:6513 hsa:2023 |
| hsa03460 | Fanconi anemia pathway | 2\|253 | 74\|9228 | 0.606644967033613 | 0.919609076442582 | ENSG00000154920 ENSG00000221829 | EME1 FANCG | hsa:146956 hsa:2189 |
| hsa04014 | Ras signaling pathway | 7\|253 | 269\|9228 | 0.610069445576039 | 0.919609076442582 | ENSG00000160867 ENSG00000152689 ENSG00000128052 ENSG00000127588 ENSG00000037280 ENSG00000143344 ENSG00000188257 | FGFR4 RASGRP3 KDR GNG13 FLT4 RGL1 PLA2G2A | hsa:2264 hsa:25780 hsa:3791 hsa:51764 hsa:2324 hsa:23179 hsa:5320 |
| hsa04390 | Hippo signaling pathway | 5\|253 | 193\|9228 | 0.614252566359872 | 0.919609076442582 | ENSG00000118523 ENSG00000092969 ENSG00000109321 ENSG00000089685 ENSG00000023445 | CTGF TGFB2 AREG BIRC5 BIRC3 | hsa:1490 hsa:7042 hsa:374 hsa:332 hsa:330 |
| hsa04261 | Adrenergic signaling in cardiomyocytes | 4\|253 | 155\|9228 | 0.6187981694859 | 0.919609076442582 | ENSG00000157613 ENSG00000171791 ENSG00000164742 novel.600 | CREB3L1 BCL2 ADCY1 - | hsa:90993 hsa:596 hsa:107 hsa:1388 |
| hsa04380 | Osteoclast differentiation | 3\|253 | 116\|9228 | 0.620978774399754 | 0.919609076442582 | ENSG00000092969 ENSG00000116701 ENSG00000132170 | TGFB2 NCF2 PPARG | hsa:7042 hsa:4688 hsa:5468 |
| hsa04931 | Insulin resistance | 3\|253 | 116\|9228 | 0.620978774399754 | 0.919609076442582 | ENSG00000157613 ENSG00000109819 ENSG00000117394 | CREB3L1 PPARGC1A SLC2A1 | hsa:90993 hsa:10891 hsa:6513 |
| hsa04662 | B cell receptor signaling pathway | 2\|253 | 76\|9228 | 0.621218272921254 | 0.919609076442582 | ENSG00000152689 ENSG00000070190 | RASGRP3 DAPP1 | hsa:25780 hsa:27071 |
| hsa00512 | Mucin type O-glycan biosynthesis | 1\|253 | 35\|9228 | 0.622732729699217 | 0.919609076442582 | ENSG00000144278 | GALNT13 | hsa:114805 |
| hsa05134 | Legionellosis | 3\|253 | 117\|9228 | 0.626791543159677 | 0.919609076442582 | ENSG00000169429 ENSG00000081041 ENSG00000137462 | CXCL8 CXCL2 TLR2 | hsa:3576 hsa:2920 hsa:7097 |
| hsa05223 | Non-small cell lung cancer | 2\|253 | 77\|9228 | 0.628347932751183 | 0.919609076442582 | novel.507 ENSG00000101412 | - E2F1 | hsa:1870 hsa:1869 |
| hsa00062 | Fatty acid elongation | 1\|253 | 36\|9228 | 0.633115479550854 | 0.919609076442582 | ENSG00000097021 | ACOT7 | hsa:11332 |
| hsa03020 | RNA polymerase | 1\|253 | 36\|9228 | 0.633115479550854 | 0.919609076442582 | ENSG00000105258 | POLR2I | hsa:5438 |
| hsa05020 | Prion diseases | 1\|253 | 38\|9228 | 0.653034818970701 | 0.941380073408789 | ENSG00000135862 | LAMC1 | hsa:3915 |
| hsa04740 | Olfactory transduction | 3\|253 | 123\|9228 | 0.660377116743596 | 0.941380073408789 | ENSG00000185532 ENSG00000127588 ENSG00000116741 | PRKG1 GNG13 RGS2 | hsa:5592 hsa:51764 hsa:5997 |
| hsa05322 | Systemic lupus erythematosus | 3\|253 | 123\|9228 | 0.660377116743596 | 0.941380073408789 | ENSG00000184260 ENSG00000124635 ENSG00000218690 | HIST2H2AC HIST1H2BJ HIST1H2APS4 | hsa:8338 hsa:8970 hsa:8336 |
| hsa00051 | Fructose and mannose metabolism | 1\|253 | 39\|9228 | 0.662586743976186 | 0.941380073408789 | ENSG00000138030 | KHK | hsa:3795 |
| hsa04068 | FoxO signaling pathway | 4\|253 | 165\|9228 | 0.667525577142486 | 0.943242663353513 | ENSG00000166851 ENSG00000092969 ENSG00000139112 ENSG00000157456 | PLK1 TGFB2 GABARAPL1 CCNB2 | hsa:5347 hsa:7042 hsa:23710 hsa:9133 |
| hsa04975 | Fat digestion and absorption | 1\|253 | 40\|9228 | 0.671876716092197 | 0.944259168562007 | ENSG00000188257 | PLA2G2A | hsa:5320 |
| hsa04620 | Toll-like receptor signaling pathway | 2\|253 | 85\|9228 | 0.681665170431581 | 0.951581172892701 | ENSG00000169429 ENSG00000137462 | CXCL8 TLR2 | hsa:3576 hsa:7097 |
| hsa04136 | Autophagy - other | 1\|253 | 42\|9228 | 0.689699231290454 | 0.951581172892701 | ENSG00000139112 | GABARAPL1 | hsa:23710 |
| hsa04010 | MAPK signaling pathway | 8\|253 | 331\|9228 | 0.692484921052549 | 0.951581172892701 | ENSG00000160867 ENSG00000152689 ENSG00000128052 ENSG00000130829 ENSG00000092969 ENSG00000109321 ENSG00000037280 ENSG00000120875 | FGFR4 RASGRP3 KDR DUSP9 TGFB2 AREG FLT4 DUSP4 | hsa:2264 hsa:25780 hsa:3791 hsa:1852 hsa:7042 hsa:374 hsa:2324 hsa:1846 |
| hsa05214 | Glioma | 2\|253 | 87\|9228 | 0.69398082766303 | 0.951581172892701 | novel.507 ENSG00000101412 | - E2F1 | hsa:1870 hsa:1869 |
| hsa04971 | Gastric acid secretion | 2\|253 | 88\|9228 | 0.699990305840345 | 0.951581172892701 | ENSG00000164742 ENSG00000147606 | ADCY1 SLC26A7 | hsa:107 hsa:115111 |
| hsa05160 | Hepatitis C | 4\|253 | 173\|9228 | 0.703335410914287 | 0.951581172892701 | novel.507 ENSG00000101412 ENSG00000111335 ENSG00000134321 | - E2F1 OAS2 RSAD2 | hsa:1870 hsa:1869 hsa:4939 hsa:91543 |
| hsa05216 | Thyroid cancer | 1\|253 | 44\|9228 | 0.706557308021646 | 0.951581172892701 | ENSG00000132170 | PPARG | hsa:5468 |
| hsa04659 | Th17 cell differentiation | 2\|253 | 91\|9228 | 0.717434948710751 | 0.951581172892701 | ENSG00000131759 ENSG00000104998 | RARA IL27RA | hsa:5914 hsa:9466 |
| hsa05120 | Epithelial cell signaling in Helicobacter pylori infection | 2\|253 | 91\|9228 | 0.717434948710751 | 0.951581172892701 | ENSG00000169429 ENSG00000081041 | CXCL8 CXCL2 | hsa:3576 hsa:2920 |
| hsa00620 | Pyruvate metabolism | 1\|253 | 46\|9228 | 0.7225029395186 | 0.951581172892701 | ENSG00000137124 | ALDH1B1 | hsa:219 |
| hsa04137 | Mitophagy - animal | 4\|253 | 180\|9228 | 0.732327906575035 | 0.951581172892701 | ENSG00000254337 ENSG00000101412 ENSG00000139112 ENSG00000123240 | AC083967.1 E2F1 GABARAPL1 OPTN | hsa:55626 hsa:1869 hsa:23710 hsa:10133 |
| hsa04750 | Inflammatory mediator regulation of TRP channels | 2\|253 | 96\|9228 | 0.744615992515707 | 0.951581172892701 | ENSG00000164742 ENSG00000168398 | ADCY1 BDKRB2 | hsa:107 hsa:624 |
| hsa00071 | Fatty acid degradation | 1\|253 | 49\|9228 | 0.744817452135621 | 0.951581172892701 | ENSG00000137124 | ALDH1B1 | hsa:219 |
| hsa01521 | EGFR tyrosine kinase inhibitor resistance | 2\|253 | 97\|9228 | 0.74977659972864 | 0.951581172892701 | ENSG00000128052 ENSG00000171791 | KDR BCL2 | hsa:3791 hsa:596 |
| hsa00514 | Other types of O-glycan biosynthesis | 1\|253 | 50\|9228 | 0.751851027101269 | 0.951581172892701 | ENSG00000144278 | GALNT13 | hsa:114805 |
| hsa05230 | Central carbon metabolism in cancer | 2\|253 | 98\|9228 | 0.754847626803226 | 0.951581172892701 | ENSG00000141526 ENSG00000117394 | SLC16A3 SLC2A1 | hsa:9123 hsa:6513 |
| hsa00565 | Ether lipid metabolism | 1\|253 | 51\|9228 | 0.758691481464244 | 0.951581172892701 | ENSG00000188257 | PLA2G2A | hsa:5320 |
| hsa04550 | Signaling pathways regulating pluripotency of stem cells | 3\|253 | 144\|9228 | 0.760523082215277 | 0.951581172892701 | ENSG00000160867 ENSG00000130829 ENSG00000105991 | FGFR4 DUSP9 HOXA1 | hsa:2264 hsa:1852 hsa:3198 |
| hsa04722 | Neurotrophin signaling pathway | 3\|253 | 145\|9228 | 0.764635371604533 | 0.951581172892701 | ENSG00000007264 ENSG00000111348 ENSG00000171791 | MATK ARHGDIB BCL2 | hsa:4145 hsa:397 hsa:596 |
| hsa00970 | Aminoacyl-tRNA biosynthesis | 1\|253 | 52\|9228 | 0.765344097263475 | 0.951581172892701 | novel.282 | - | hsa:54938 |
| hsa05203 | Viral carcinogenesis | 6\|253 | 275\|9228 | 0.770791486481744 | 0.951581172892701 | ENSG00000157613 ENSG00000179388 ENSG00000117399 ENSG00000124635 ENSG00000145386 novel.600 | CREB3L1 EGR3 CDC20 HIST1H2BJ CCNA2 - | hsa:90993 hsa:1960 hsa:991 hsa:8970 hsa:890 hsa:1388 |
| hsa04670 | Leukocyte transendothelial migration | 3\|253 | 147\|9228 | 0.772688587309783 | 0.951581172892701 | ENSG00000107562 ENSG00000179776 ENSG00000116701 | CXCL12 CDH5 NCF2 | hsa:6387 hsa:1003 hsa:4688 |
| hsa05130 | Pathogenic Escherichia coli infection | 6\|253 | 276\|9228 | 0.773762838347188 | 0.951581172892701 | ENSG00000169429 ENSG00000214222 ENSG00000123416 ENSG00000109062 ENSG00000006453 ENSG00000104833 | CXCL8 TUBBP2 TUBA1B SLC9A3R1 BAIAP2L1 TUBB4A | hsa:3576 hsa:203068 hsa:10376 hsa:9368 hsa:55971 hsa:10382 |
| hsa04919 | Thyroid hormone signaling pathway | 3\|253 | 148\|9228 | 0.776630327707052 | 0.951581172892701 | ENSG00000159200 ENSG00000117394 ENSG00000211448 | RCAN1 SLC2A1 DIO2 | hsa:1827 hsa:6513 hsa:1734 |
| hsa00140 | Steroid hormone biosynthesis | 1\|253 | 54\|9228 | 0.778106225686569 | 0.951581172892701 | ENSG00000101846 | STS | hsa:412 |
| hsa00380 | Tryptophan metabolism | 1\|253 | 54\|9228 | 0.778106225686569 | 0.951581172892701 | ENSG00000137124 | ALDH1B1 | hsa:219 |
| hsa04520 | Adherens junction | 2\|253 | 103\|9228 | 0.778894803434968 | 0.951581172892701 | ENSG00000173482 ENSG00000127329 | PTPRM PTPRB | hsa:5797 hsa:5787 |
| hsa04912 | GnRH signaling pathway | 2\|253 | 104\|9228 | 0.783449934860427 | 0.951581172892701 | ENSG00000135346 ENSG00000164742 | CGA ADCY1 | hsa:1081 hsa:107 |
| hsa00250 | Alanine, aspartate and glutamate metabolism | 1\|253 | 55\|9228 | 0.784225598359481 | 0.951581172892701 | ENSG00000086205 | FOLH1 | hsa:2346 |
| hsa04730 | Long-term depression | 1\|253 | 56\|9228 | 0.790176860063945 | 0.951581172892701 | ENSG00000185532 | PRKG1 | hsa:5592 |
| hsa05164 | Influenza A | 4\|253 | 196\|9228 | 0.790544359018552 | 0.951581172892701 | ENSG00000137648 ENSG00000169429 ENSG00000111335 ENSG00000134321 | TMPRSS4 CXCL8 OAS2 RSAD2 | hsa:56649 hsa:3576 hsa:4939 hsa:91543 |
| hsa05412 | Arrhythmogenic right ventricular cardiomyopathy (ARVC) | 2\|253 | 107\|9228 | 0.796625783744002 | 0.954482505868389 | ENSG00000005961 ENSG00000115221 | ITGA2B ITGB6 | hsa:3674 hsa:3694 |
| hsa04340 | Hedgehog signaling pathway | 1\|253 | 58\|9228 | 0.801593327187688 | 0.956028738847701 | ENSG00000171791 | BCL2 | hsa:596 |
| hsa04330 | Notch signaling pathway | 1\|253 | 60\|9228 | 0.812390946547007 | 0.957512310249372 | ENSG00000135547 | HEY2 | hsa:23493 |
| hsa04666 | Fc gamma R-mediated phagocytosis | 2\|253 | 112\|9228 | 0.817018062546169 | 0.957512310249372 | ENSG00000078053 ENSG00000277443 | AMPH MARCKS | hsa:273 hsa:4082 |
| hsa05100 | Bacterial invasion of epithelial cells | 2\|253 | 112\|9228 | 0.817018062546169 | 0.957512310249372 | ENSG00000197959 ENSG00000116670 | DNM3 MAD2L2 | hsa:26052 hsa:10459 |
| hsa04622 | RIG-I-like receptor signaling pathway | 1\|253 | 61\|9228 | 0.817568203366772 | 0.957512310249372 | ENSG00000169429 | CXCL8 | hsa:3576 |
| hsa05014 | Amyotrophic lateral sclerosis (ALS) | 1\|253 | 62\|9228 | 0.822603137865322 | 0.959088860291406 | ENSG00000171791 | BCL2 | hsa:596 |
| hsa04072 | Phospholipase D signaling pathway | 3\|253 | 165\|9228 | 0.835444681831966 | 0.968875516322406 | ENSG00000197959 ENSG00000169429 ENSG00000164742 | DNM3 CXCL8 ADCY1 | hsa:26052 hsa:3576 hsa:107 |
| hsa04723 | Retrograde endocannabinoid signaling | 3\|253 | 166\|9228 | 0.838449966048236 | 0.968875516322406 | ENSG00000127588 ENSG00000187730 ENSG00000164742 | GNG13 GABRD ADCY1 | hsa:51764 hsa:2563 hsa:107 |
| hsa04370 | VEGF signaling pathway | 1\|253 | 69\|9228 | 0.854176992263097 | 0.974061482405286 | ENSG00000128052 | KDR | hsa:3791 |
| hsa04924 | Renin secretion | 1\|253 | 69\|9228 | 0.854176992263097 | 0.974061482405286 | ENSG00000078401 | EDN1 | hsa:1906 |
| hsa05133 | Pertussis | 1\|253 | 69\|9228 | 0.854176992263097 | 0.974061482405286 | ENSG00000169429 | CXCL8 | hsa:3576 |
| hsa04720 | Long-term potentiation | 1\|253 | 72\|9228 | 0.865931760896987 | 0.978811481927082 | ENSG00000164742 | ADCY1 | hsa:107 |
| hsa04140 | Autophagy - animal | 5\|253 | 270\|9228 | 0.868042902509374 | 0.978811481927082 | ENSG00000254337 ENSG00000171791 ENSG00000139112 ENSG00000078804 ENSG00000062716 | AC083967.1 BCL2 GABARAPL1 TP53INP2 VMP1 | hsa:55626 hsa:596 hsa:23710 hsa:58476 hsa:81671 |
| hsa04978 | Mineral absorption | 1\|253 | 73\|9228 | 0.869636355096754 | 0.978811481927082 | ENSG00000198417 | MT1F | hsa:4494 |
| hsa04921 | Oxytocin signaling pathway | 3\|253 | 180\|9228 | 0.875798937252708 | 0.981498808990104 | ENSG00000159200 ENSG00000116741 ENSG00000164742 | RCAN1 RGS2 ADCY1 | hsa:1827 hsa:5997 hsa:107 |
| hsa04213 | Longevity regulating pathway - multiple species | 1\|253 | 77\|9228 | 0.88346258821539 | 0.981625098017101 | ENSG00000164742 | ADCY1 | hsa:107 |
| hsa04658 | Th1 and Th2 cell differentiation | 1\|253 | 77\|9228 | 0.88346258821539 | 0.981625098017101 | ENSG00000138378 | STAT4 | hsa:6775 |
| hsa05205 | Proteoglycans in cancer | 4\|253 | 238\|9228 | 0.896229759476093 | 0.985533193535438 | ENSG00000128052 ENSG00000092969 ENSG00000090447 ENSG00000137462 | KDR TGFB2 TFAP4 TLR2 | hsa:3791 hsa:7042 hsa:7023 hsa:7097 |
| hsa05170 | Human immunodeficiency virus 1 infection | 4\|253 | 242\|9228 | 0.903319350003694 | 0.985533193535438 | ENSG00000127588 ENSG00000171791 ENSG00000137462 ENSG00000157456 | GNG13 BCL2 TLR2 CCNB2 | hsa:51764 hsa:596 hsa:7097 hsa:9133 |
| hsa04721 | Synaptic vesicle cycle | 1\|253 | 87\|9228 | 0.911967494404423 | 0.985533193535438 | ENSG00000197959 | DNM3 | hsa:26052 |
| hsa04144 | Endocytosis | 6\|253 | 343\|9228 | 0.913886708227422 | 0.985533193535438 | ENSG00000160867 ENSG00000078053 ENSG00000197959 ENSG00000153071 ENSG00000114423 ENSG00000024422 | FGFR4 AMPH DNM3 DAB2 CBLB EHD2 | hsa:2264 hsa:273 hsa:26052 hsa:1601 hsa:868 hsa:30846 |
| hsa04530 | Tight junction | 3\|253 | 200\|9228 | 0.915906144732153 | 0.985533193535438 | ENSG00000123416 ENSG00000166025 ENSG00000109062 | TUBA1B AMOTL1 SLC9A3R1 | hsa:10376 hsa:154810 hsa:9368 |
| hsa04020 | Calcium signaling pathway | 3\|253 | 202\|9228 | 0.919190993614026 | 0.985533193535438 | ENSG00000168539 ENSG00000164742 ENSG00000168398 | CHRM1 ADCY1 BDKRB2 | hsa:1128 hsa:107 hsa:624 |
| hsa00230 | Purine metabolism | 2\|253 | 153\|9228 | 0.926110952041713 | 0.985533193535438 | ENSG00000168032 ENSG00000164742 | ENTPD3 ADCY1 | hsa:956 hsa:107 |
| hsa04141 | Protein processing in endoplasmic reticulum | 3\|253 | 210\|9228 | 0.931195109714094 | 0.985533193535438 | ENSG00000162909 ENSG00000171791 novel.600 | CAPN2 BCL2 - | hsa:824 hsa:596 hsa:1388 |
| hsa04660 | T cell receptor signaling pathway | 1\|253 | 98\|9228 | 0.935362830564239 | 0.985533193535438 | ENSG00000114423 | CBLB | hsa:868 |
| hsa05235 | PD-L1 expression and PD-1 checkpoint pathway in cancer | 1\|253 | 99\|9228 | 0.937153981042579 | 0.985533193535438 | ENSG00000137462 | TLR2 | hsa:7097 |
| hsa05132 | Salmonella infection | 5\|253 | 317\|9228 | 0.939408561383005 | 0.985533193535438 | ENSG00000169429 ENSG00000171791 ENSG00000023445 ENSG00000137462 ENSG00000233328 | CXCL8 BCL2 BIRC3 TLR2 PFN1P1 | hsa:3576 hsa:596 hsa:330 hsa:7097 hsa:5216 |
| hsa04142 | Lysosome | 2\|253 | 162\|9228 | 0.939924015456304 | 0.985533193535438 | ENSG00000119899 ENSG00000102393 | SLC17A5 GLA | hsa:26503 hsa:2717 |
| hsa05135 | Yersinia infection | 2\|253 | 164\|9228 | 0.942642875937608 | 0.985533193535438 | ENSG00000169429 ENSG00000082074 | CXCL8 FYB1 | hsa:3576 hsa:2533 |
| hsa04350 | TGF-beta signaling pathway | 1\|253 | 103\|9228 | 0.943837558424323 | 0.985533193535438 | ENSG00000092969 | TGFB2 | hsa:7042 |
| hsa04726 | Serotonergic synapse | 1\|253 | 103\|9228 | 0.943837558424323 | 0.985533193535438 | ENSG00000127588 | GNG13 | hsa:51764 |
| hsa04310 | Wnt signaling pathway | 2\|253 | 169\|9228 | 0.948938837473335 | 0.986544807906339 | ENSG00000133067 ENSG00000175792 | LGR6 RUVBL1 | hsa:59352 hsa:8607 |
| hsa04910 | Insulin signaling pathway | 2\|253 | 172\|9228 | 0.952395179940351 | 0.986544807906339 | ENSG00000109819 ENSG00000114423 | PPARGC1A CBLB | hsa:10891 hsa:868 |
| hsa01200 | Carbon metabolism | 2\|253 | 183\|9228 | 0.963256883030527 | 0.991136168610261 | ENSG00000186197 ENSG00000092621 | EDARADD PHGDH | hsa:2023 hsa:26227 |
| hsa03013 | RNA transport | 3\|253 | 246\|9228 | 0.967475691907627 | 0.991136168610261 | novel.674 ENSG00000013810 ENSG00000183684 | - TACC3 ALYREF | hsa:79897 hsa:10460 hsa:10189 |
| hsa03015 | mRNA surveillance pathway | 1\|253 | 126\|9228 | 0.970607183068367 | 0.991136168610261 | ENSG00000183684 | ALYREF | hsa:10189 |
| hsa04714 | Thermogenesis | 5\|253 | 360\|9228 | 0.972075857675448 | 0.991136168610261 | ENSG00000185532 ENSG00000157613 ENSG00000109819 ENSG00000164742 ENSG00000132170 | PRKG1 CREB3L1 PPARGC1A ADCY1 PPARG | hsa:5592 hsa:90993 hsa:10891 hsa:107 hsa:5468 |
| hsa05131 | Shigellosis | 5\|253 | 375\|9228 | 0.978944192158419 | 0.994240195160894 | ENSG00000169429 ENSG00000162909 ENSG00000171791 ENSG00000139112 ENSG00000233328 | CXCL8 CAPN2 BCL2 GABARAPL1 PFN1P1 | hsa:3576 hsa:824 hsa:596 hsa:23710 hsa:5216 |
| hsa03040 | Spliceosome | 2\|253 | 229\|9228 | 0.987916545860286 | 0.998841971703493 | ENSG00000130520 ENSG00000183684 | LSM4 ALYREF | hsa:25804 hsa:10189 |
| hsa05012 | Parkinson disease | 4\|253 | 430\|9228 | 0.997924217739206 | 0.998841971703493 | ENSG00000214222 ENSG00000123416 ENSG00000180176 ENSG00000104833 | TUBBP2 TUBA1B TH TUBB4A | hsa:203068 hsa:10376 hsa:7054 hsa:10382 |
| hsa04932 | Non-alcoholic fatty liver disease (NAFLD) | 1\|253 | 225\|9228 | 0.998223546920143 | 0.998841971703493 | ENSG00000169429 | CXCL8 | hsa:3576 |
| hsa05017 | Spinocerebellar ataxia | 1\|253 | 240\|9228 | 0.998841971703493 | 0.998841971703493 | ENSG00000254337 | AC083967.1 | hsa:55626 |
